# Elevated MARCKS phosphorylation contributes to unresponsiveness of breast cancer to paclitaxel treatment

**DOI:** 10.18632/oncotarget.3827

**Published:** 2015-04-14

**Authors:** Ching-Hsien Chen, Chun-Ting Cheng, Yuan Yuan, Jing Zhai, Muhammad Arif, Lon Wolf R. Fong, Reen Wu, David K. Ann

**Affiliations:** ^1^ Department of Internal Medicine, Division of Pulmonary and Critical Care Medicine and Center for Comparative Respiratory Biology and Medicine, University of California Davis, California, USA; ^2^ Department of Molecular Pharmacology, Beckman Research Institute, City of Hope, Duarte, California, USA; ^3^ Irell and Manella Graduate School of Biological Sciences, Beckman Research Institute, City of Hope, Duarte, California, USA; ^4^ Department of Medical Oncology and Experimental Therapeutics, City of Hope Comprehensive Cancer Center, Duarte, California, USA; ^5^ Department of Pathology, City of Hope Comprehensive Cancer Center, Duarte, California, USA

**Keywords:** phospho-MARCKS, MANS peptide, paclitaxel, mitotic inhibitor, breast cancer

## Abstract

Accumulating evidence has suggested that myristoylated alanine-rich C-kinase substrate (MARCKS) is critical for regulating multiple pathophysiological processes. However, the molecular mechanism underlying increased phosphorylation of MARCKS at Ser159/163 (phospho-MARCKS) and its functional consequence in neoplastic disease remain to be established. Herein, we investigated how phospho-MARCKS is regulated in breast carcinoma, and its role in the context of chemotherapy. In a screen of patients with breast tumors, we find that the abundance of phospho-MARCKS, not MARCKS protein *per se*, increased in breast cancers and positively correlated with tumor grade and metastatic status. Among chemotherapeutic agents, mitotic inhibitors, including paclitaxel, vincristine or eribulin, notably promoted phospho-MARCKS accumulation in multiple breast cancer cells. We further show that phospho-MARCKS acted upstream of Src activation upon paclitaxel exposure. Reduction of phospho-MARCKS by knockdown of MARCKS or pharmacological agents increased paclitaxel sensitivity. Particularly, a known phospho-MARCKS inhibitor, MANS peptide, was demonstrated to increase paclitaxel efficacy and attenuate angiogenesis/metastasis of xenografted breast cancer cells by decreasing abundance of phospho-MARCKS and messages of inflammatory mediators. Our data suggest that unresponsiveness of breast cancer to paclitaxel treatment is, at least in part, mediated by phospho-MARCKS and also provide an alternative therapeutic strategy against breast cancer by improving taxanes sensitivity.

## INTRODUCTION

Cytotoxic chemotherapy remains a mainstay for breast cancer treatment, especially for recurrent or metastatic breast cancer (MBC) [1]. Mitotic inhibitors are one class of chemotherapeutic agents commonly used in cancer treatment; they interfere with the dynamic instability of spindle microtubules, most by binding reversibly to different sites on β-tubulin, leading to arrest of the cell cycle and subsequent apoptosis. There are two main modes of action within mitotic inhibitors: 1) taxanes (which include paclitaxel and docetaxel) stabilize microtubules and promote polymerization; 2) vinca alkaloids and the newly approved eribulin destabilize microtubules and prevent growth [2, 3]. Among these agents, the taxanes have served as a standard first-line option for triple-negative breast cancer (TNBC) and MBC. However, the prevalence of taxanes in early-stage treatment has contributed to the development of chemoresistance at later stages of the disease [4-6]. A direct comparison of paclitaxel treatment in 585 breast cancer patients, single-agent paclitaxel as a chemotherapeutic regimen for MBC demonstrated lower response rates both in weekly and 3-weekly administrations (40% and 28%) [7]. This poor response to chemotherapy has created challenges in clinical practice; and there is an urgent need to identify a predictive marker to identify responders and non-responders.

Dysfunctional apoptotic signaling attributes to, at least in part, cellular resistance to chemotherapeutic agents. Some signaling events, which minimize chemotherapy efficacy and/or promote metastatic development, are induced following the exposure to chemotherapy [8]. For instance, Src-family kinases (SFKs), a family of non-receptor tyrosine kinases, have been implicated promoting paclitaxel resistance by activating survival pathways, attenuating autophagy, and modulating angiogenesis [9, 10]. Paclitaxel in particular has also been reported to enhance epithelial-mesenchymal transition and cell motility as well as invasive ability [11]. Tumor cells also survive the chemotherapy through increased production of cytokines, chemokines, and growth factors via paracrine or autocrine [12]. Several studies have demonstrated up-regulation of IL-1β, IL-6, IL-8, and vascular endothelial growth factor (VEGF) in response to paclitaxel treatment [13-17].

Myristoylated alanine-rich C-kinase substrate (MARCKS), predominantly those forms phosphorylated at Ser159 and Ser163 (phospho-MARCKS), has been extensively studied in inflammatory disease related to both influx of inflammatory cells into the lung and release of inflammatory mediators by these cells [18]. In particular, a pharmacological inhibitor of MARCKS, termed MANS peptide, is known to be capable of inhibiting cell motility, mucus hypersecretion, and cytokine expression [19-22]. Recently, increasing evidence points to phospho-MARCKS as a potential target in cancer progression [23-26] and our laboratory also discovered its significance in lung cancer [27-30]. However, the regulation of phospho-MARCKS abundance and its functional consequence in breast cancer are yet to be elucidated.

Metastasis and resistance to chemotherapy account for the majority of causes of treatment failure; thus, we have three aims in this study: 1) to establish clinical relevance of phospho-MARCKS in breast cancer; 2) to characterize the functional roles of phospho-MARCKS in response to chemotherapy *in vitro*; and 3) to determine whether pharmacological inhibition of MARCKS by the MANS peptide improves paclitaxel efficacy *in vivo*. Our studies point to the critical role of phospho-MARCKS in the unresponsiveness of breast cancer to taxanes and suggest that phospho-MARCKS is a druggable target to overcome resistance to paclitaxel.

## RESULTS

### Increased MARCKS phosphorylation is associated with metastatic potential of breast cancer

**Figure 1:**
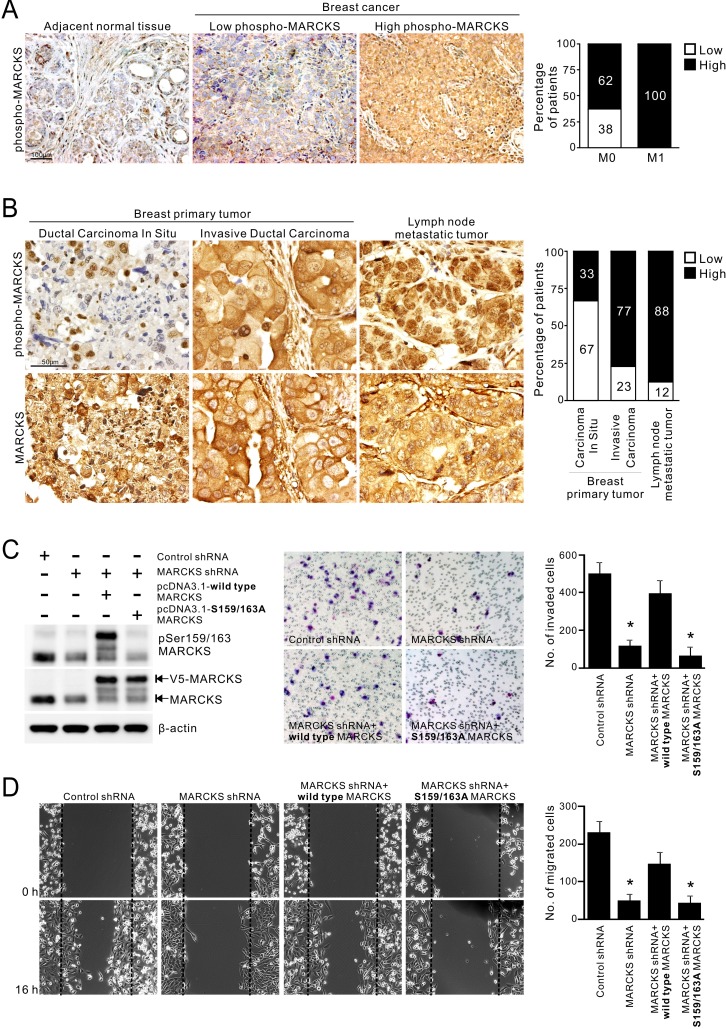
Elevated phospho-MARCKS abundance in invasive breast cancer **A.** Correlation of high phospho-MARCKS levels with distant metastasis of breast cancer. *Left*, representative images of immunohistochemical (IHC) staining in adjacent non-tumor areas and breast cancer specimens with low levels of phospho-MARCKS (score = +1 or +2) and high phospho-MARCKS abundance (score = +3). *Right*, percentage of patients with high and low phospho-MARCKS according to no distant metastasis (M0) vs distant metastasis (M1). *P* = 0.042, Fisher's exact test. **B.** *Left*, representative IHC images of breast primary tumors and lymph node metastatic tumors by using anti-phospho-MARCKS and anti-MARCKS antibodies. *Right*, percentage of patients with high and low phospho-MARCKS according to cancer types (noninvasive ductal carcinomas in situ, invasive breast carcinomas and lymph node metastatic tumors). *p* = 0.048, Fisher's exact test. (C-D) Higher phospho-MARCKS promotes breast cancer cell invasion and migration. MDA-MB-231 cells were transduced with control non-specific or MARCKS-specific shRNA-containing lentiviruses. Following knockdown of MARCKS, cells were re-expressed either wild type or mutant (S159/163A) V5-tagged MARCKS. **C.** *Left,* the levels of phospho-MARCKS abundance and MARCKS expression in these genetically modified cells were determined by Western blot. These cells were plated on Transwells with matrigel; 20 hours later, migrated cells were fixed, stained, and counted using light microscopy. A representative picture of each group is shown in the *middle*. *Right*, quantification of migrated cells to the lower chamber. Data expressed as mean ± SD (*n* = 4), *p < 0.05 as compared to cells receiving control shRNA. **D.** Confluent cultures of these cells were scratched and wound healing repair was monitored microscopically after the scratch. *Left*, representative phase contrast pictures. *Right*, numbers of cells migrated to the wound area were quantified at 16 hours post-scratching. (*n* = 4, *p < 0.05 versus control shRNA).

To investigate the role of phospho-MARCKS (Ser159/163) in breast cancer, we evaluated phospho-MARCKS abundance by IHC analysis in primary tumor tissue sections from 21 patients with breast carcinomas. The IHC results were classified into two groups according to the intensity and extent of staining, as described in “Methods”. Samples that were strong positive (score = +3) were defined as high phospho-MARCKS. Comparing to adjacent normal breast tissues, high phospho-MARCKS was detected in 86% (*n* = 18/21) of breast tumor specimens (Figure 1A, *left*). Strikingly, abundant phospho-MARCKS staining in samples was significantly greater in patients with distant metastases (M1) compared to patients with no distant metastases (M0), at a rate of 100% (*n* = 13/13) versus 62% (*n* = 5/8) (Figure 1A, *right; p* = 0.042). To ascertain whether phospho-MARCKS is associated with invasive/metastatic potential of breast cancer, we then validated the phospho-MARCKS signal abundance in another cohort of patients (*n* = 50) with various types of breast tumors, including primary and lymph node metastatic tumors (Figure 1B, *left* and Supplementary Table S1). Interestingly, phospho-MARCKS signals were significantly lower in noninvasive tumors compared to those invasive breast carcinomas and lymph node metastatic tumors (Figure 1B, *right*; *p* = 0.048). A positive correlation between tumor grade and phospho-MARCKS was established (Supplementary Table S1; *p* = 0.005). However, there was no significant association of total MARCKS abundance with aggressive phenotype.

We next assessed phospho-MARCKS and total MARCKS abundance in some breast cancer cell lines (Supplementary Figure S1). Western blots demonstrated that both phospho-MARCKS and MARCKS expressions were higher in the invasive breast cancer cell lines, MDA-MB-231 and MDA-MB-468 [31]. To determine whether an increase in phospho-MARCKS or total MARCKS abundance promotes breast cancer cell invasiveness and motility, we used a MARCKS-specific short hairpin RNA (MARCKS-shRNA) to deplete endogenous MARCKS, then followed by re-expression of wild-type or S159/163A-MARCKS. As shown in Figures 1C and 1D, silencing MARCKS expression in high MARCKS-expressing MDA-MB-231 cells resulted in reducing cancer invasion and migration as compared to control shRNA-transduced cells. Whereas reconstituted wild-type MARCKS restored invasive phenotype, but phosphorylation-defective S159/163A-MARCKS failed. Altogether, our results supported a critical role for phospho-MARCKS in mediating breast cancer cell invasion and migration.

### Implications of phospho-MARCKS levels in mitotic inhibitor paclitaxel-induced cytotoxicity

**Figure 2:**
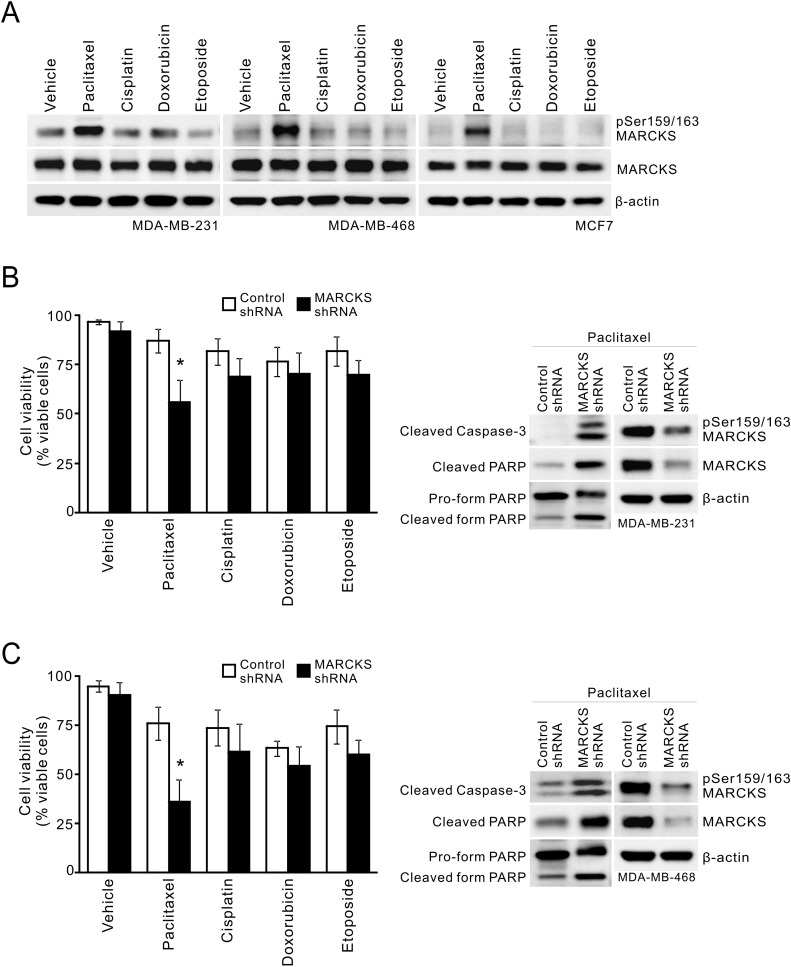
MARCKS inhibition is associated with increased paclitaxel cytotoxicity **A.** Analysis of phospho-MARCKS levels in breast cancer cells after exposure to chemotherapy. Cells were incubated with various chemotherapeutic agents including paclitaxel (20 nM), cisplatin (15 μM), doxorubicin (25 nM) and etoposide (5 μM), respectively. After 24 hours, cells were collected and subjected to Western blot analysis. **B-C.** Knockdown of MARCKS to down-regulate phospho-MARCKS levels decreases cell viability of MDA-MB-231 **B.** and MDA-MB-468 **C.** cells in response to chemotherapy. Cells were transduced with control non-specific or MARCKS-specific shRNA-containing lentiviruses. These cells were subjected to 20 nM paclitaxel, 15 μM cisplatin, 25 nM doxorubicin or 5 μM etoposide for treatment. After 72 hours, cell viability was assessed by Trypan blue staining (*left*). Data shown as mean ± SD; _*_: *p* < 0.05 versus control shRNA (*n* = 4). *Right*, Western blot analysis of cleaved caspase-3 and PARP in control shRNA and MARCKS-knockdown cells after 48 hours of treatment with 20 nM paclitaxel.

Since chemotherapy has been used to treat all stages of breast cancer, particularly MBCs [1], we examined the effect of chemotherapy treatment on phospho-MARCKS abundance. Three breast cancer cell lines were treated with different chemotherapeutic agents, including cisplatin, paclitaxel, doxorubicin or etoposide. Figure 2A shows that there was an increase in phospho-MARCKS in the cells exposed to a mitotic inhibitor, paclitaxel, over vehicle-treated counterparts, whereas no significant sensitizing effect was noted in cells treated with other chemotherapeutic agents. We next asked if elevated phospho-MARCKS abundance is associated with decreased breast cancer cell survival in response to chemotherapy. shRNA silencing approach was used to eliminate MARCKS in two TNBC cell lines with abundant phospho-MARCKS, MDA-MB-231 and MDA-MB-468 (Supplementary Figure S1). Cells were exposed to cisplatin, paclitaxel, doxorubicin or etoposide for 72 hours. Cell viability was reduced by 31% (Figure 2B, *left*) and 40% (Figure 2C, *left*), respectively, by MARCKS-shRNA, compared to that in cells receiving control-shRNA after paclitaxel treatment. Knockdown of MARCKS did not significantly enhance cytotoxicity of any additional chemotherapeutic agents examined. Likewise, data from MTS assays also supported that MARCKS-knockdown cells were more sensitive to paclitaxel (Supplementary Figure S2). Western blots revealed increased caspase-3 cleavage, hallmark of caspase-3 activation, in these silenced cells upon paclitaxel exposure (Figures 2B and 2C, *right*). Altogether, these data suggest that phospho-MARCKS abundance is inversely associated with the efficacy of paclitaxel in TNBC cells.

### Activation of PKC/MARCKS pathway in breast cancer upon mitotic inhibitors treatment

**Figure 3:**
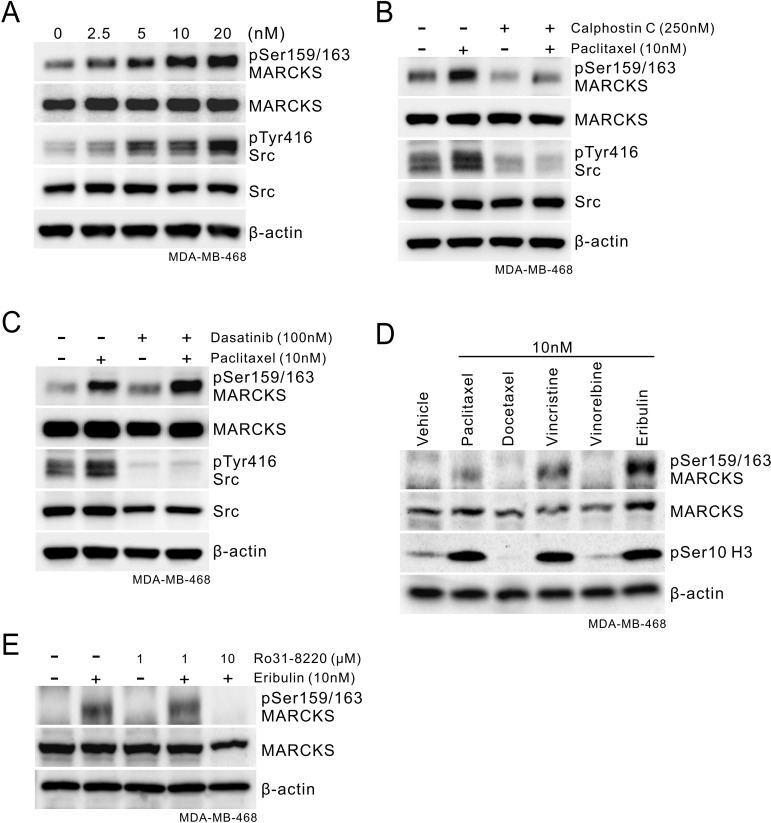
Phospho-MARCKS increases in response to mitotic inhibitors treatment **A.** Paclitaxel treatment induces activation of MARCKS and Src in TNBC cells. Cells were incubated with various doses of paclitaxel as indicated. After 24 hours, cells were collected and subjected to Western blot analysis. **B-C.** Determination of the major kinase that led to MARCKS activation in these TNBC cells in response to paclitaxel. B, cells were co-treated with paclitaxel (10 nM) and PKC inhibitor (Calphostin C; 250 nM). C, cells were co-treated with paclitaxel (10 nM) and Src inhibitor (Dasatinib; 100 nM). After 24 hours of co-treatment, Western blots were carried out with specific antibodies as indicated. **D.** Western blot analysis of phospho-MARCKS levels in MDA-MB-468 cells upon treatment with various mitotic inhibitors for 24 hours. **E.** Eribulin-treated cells were incubated with or without PKC inhibitor (Ro 31-8220) for 24 hours and then subjected to immunoblotting analysis.

To further validate the role of phospho-MARCKS in conveying paclitaxel resistance in breast cancer cells, we performed a dose-course analysis of MDA-MB-468 cells undergoing paclitaxel treatment. We showed that there was no apoptotic effect in these cells in the presence of paclitaxel at concentrations from 0 to 20 nM (Supplementary Figure S3A). Notably, phospho-MARCKS abundance increased in a concentration-dependent manner (Figure 3A), concomitant with induced Src activation, which crosstalk with mechanisms that regulate the efficacy of paclitaxel [9]. However, steady state MARCKS expression remained unchanged in paclitaxel-treated cells. A similar phenomenon was recapitulated in ER-positive MCF7 cells upon paclitaxel stimulation (Supplementary Figure S3B). To determine the major kinase that phosphorylated MARCKS in response to paclitaxel, a potent pan-PKC inhibitor (250 nM Calphostin C) was used in combination with paclitaxel to treat these cells. Western blot analyses demonstrated that Calphostin C suppressed paclitaxel-dependent up-regulation of phospho-MARCKS in all these cells (Figure 3B and Supplementary Figure S3C). In an attempt to define the specific PKC isoforms involved in paclitaxel-enhanced phospho-MARCKS, cells were preincubated with various isoform-specific PKC inhibitors for 30 minutes followed by co-incubation with paclitaxel. We found that two of the inhibitors, alpha-isoform-selective (Gö6976) and delta-isoform-selective (Rottlerin) PKC inhibitors, reduced phospho-MARCKS abundance in paclitaxel-treated cells (Supplementary Figure S3D). Next, co-treatment with a Src inhibitor (100 nM Dasatinib) was performed to examine whether the elevated phospho-MARCKS was through activated Src. Figure 3C reveals that phospho-MARCKS abundance persisted, in the presence of Src inhibitor, following the exposure to paclitaxel. Instead, knockdown of MARCKS decreased Src pTyr416 abundance in TNBC cells (Supplementary Figure S3E). To expand on the above finding, the effect of several mitotic inhibitors on phospho-MARCKS abundance was investigated. Remarkably, phospho-MARCKS was up-regulated by vincristine and eribulin in addition to paclitaxel (Figure 3D). We also observed an association between abundances of phospho-MARCKS and phospho-Histone H3 (Ser10), a surrogate marker for mitosis. Treatment with a new anti-mitotic agent, eribulin, also resulted in Ro31-8220, a pan-PKC inhibitor, -sensitive increase of phospho-MARCKS in MDA-MB-468, MDA-MB-231 and MCF7 cells (Figure 3E and Supplementary Figure S3F). These data convincingly demonstrate that phospho-MARCKS increased upon the inhibition of breast cancer cell mitosis.

### Elevated phospho-MARCKS supports unresponsiveness of breast cancer cells to paclitaxel

**Figure 4:**
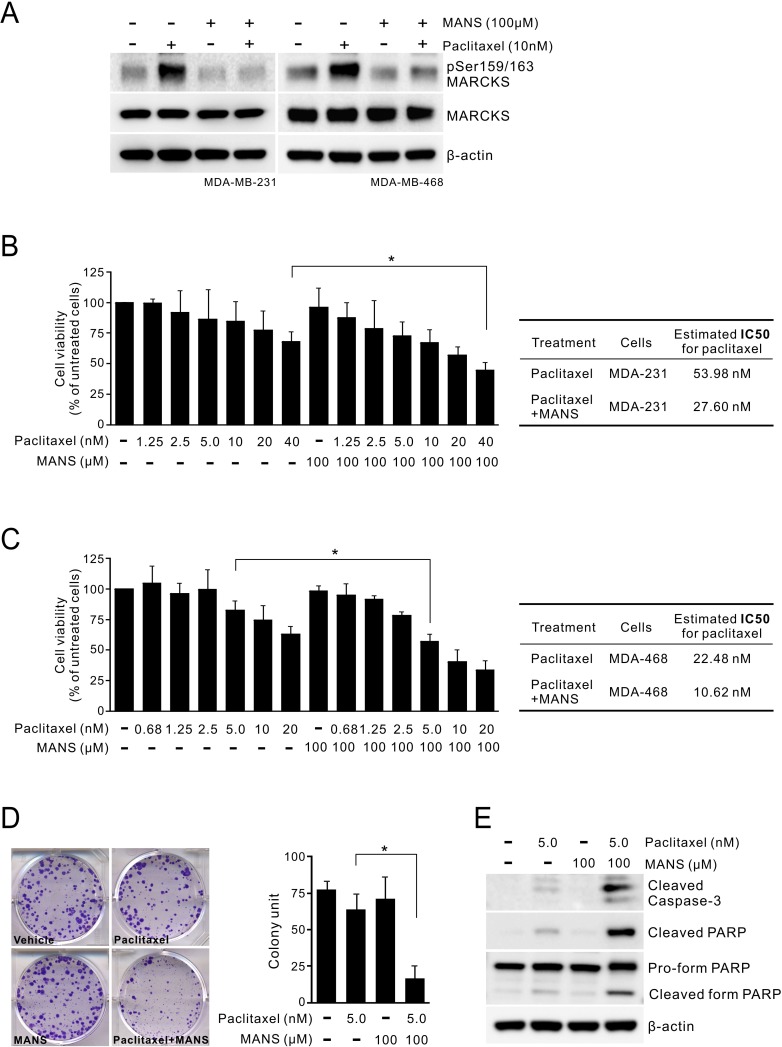
Co-treatment of MARCKS inhibitor and paclitaxel suppresses proliferation of breast cancer **A.** MARCKS inhibitor MANS peptide inhibited paclitaxel-induced MARCKS phosphorylation of breast cancer cells. MDA-MB-231 (*left*) and MDA-MB-468 (*right*) cells were pre-treated with 100 μM MANS peptide for 12 hours and then co-treated with paclitaxel or left alone as indicated. These cells were harvested 24 hours later and subjected to immunoblot analysis. (B-C) The combinatorial effect of MANS peptide with paclitaxel on breast cancer cell lines. MDA-MB-231 **B.** and MDA-MB-468 **C.**, were co-treated with various dosages of paclitaxel and 100 μM MANS peptide. After 72 hours, cell viability was determined by MTS assay. _*_: *p* < 0.05. *Right table*, estimated IC50 values for paclitaxel alone and combined treatments with MANS peptide. **D.** Cells were treated with the indicated concentrations of MANS peptide combined with paclitaxel and colonies were counted after 10 days by using crystal violet staining. *Left*, data are representative of three independent experiments. *Right*, expression of colony formation index. **E.** Caspase-3 activation in MANS-treated cells upon paclitaxel treatment. Cells were incubated in a medium containing either paclitaxel, MANS peptide or combination of paclitaxel and MANS peptide for 48 hours and then subjected to Western blot analyses.

We previously reported that the use of MANS peptide, which targets the N-terminal myristoylation site in MARCKS, was able to reduce phospho-MARCKS levels in lung cancer [27]. Through the combination treatment of breast cancer using MANS peptide and paclitaxel, we confirmed that MANS peptide has an inhibitory effect on paclitaxel-enhanced MARCKS phosphorylation, as PKC inhibitor did (Figure 4A). Since PKC participates in multiple complex signaling cascades, we used MANS peptide to specifically block the effect of phospho-MARCKS on paclitaxel resistance. MTS assays showed that the combination of paclitaxel (1.25 - 40 nM) with 100 μM MANS peptide resulted in a decrease of paclitaxel IC50 (half maximal inhibitory concentration) from 53.98 to 27.60 nM in MDA-MB-231 cells (Figure 4B). This combined effect was also noted of MDA-MB-468 cells (Figure 4C). Similarly, analysis of clonogenic abilities revealed that the co-treatment with 100 μM of MANS peptide increased the anti-proliferation activity of paclitaxel approximately 3.8-fold (Figure 4D). MANS-treated cells showed a marked elevation of cleaved caspase-3 and PARP in response to paclitaxel (Figure 4E). The results suggest that high phospho-MARCKS abundance caused by paclitaxel contributes to the resistance to paclitaxel in breast cancer cells.

### Suppression of phospho-MARCKS enhances paclitaxel sensitivity *in vivo*

**Figure 5:**
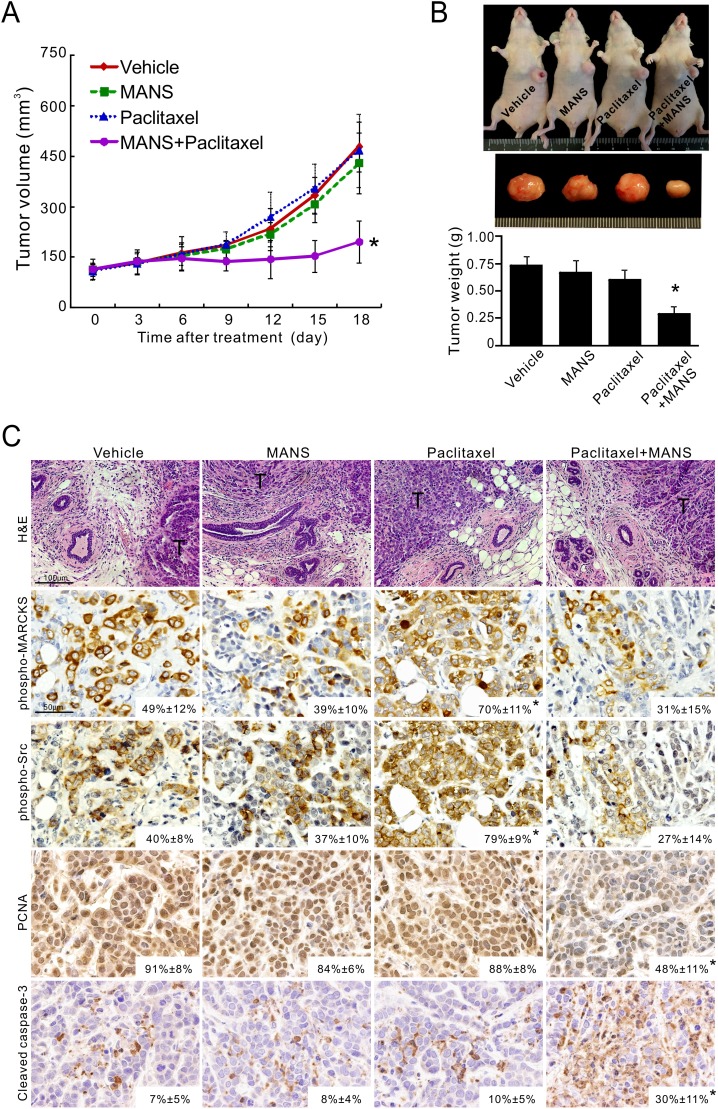
Targeting phospho-MARCKS improves paclitaxel efficiency *in vivo* **A-B.** Growth curves **A.** and tumor weights **B.** of xenograft tumors generated by orthotopic injection of MDA-MB-468 cells into the fat pads of mice breasts. Once tumor volume reached an average of 100 mm^3^ at the injected site, mice were randomly grouped for intraperitoneal injection, once every three days with either vehicle, MANS peptide (12.5 mg/kg), paclitaxel (3 mg/kg) alone or paclitaxel with MANS peptide (12.5 mg/kg). Tumor measurements were taken every three days after each drug injection and data were presented as mean ± SD (A). After 21 days of treatment, the xenograft tumors of these mice were removed and weighed. Data were expressed as the mean ± SE (B). _*_: *p* < 0.05 for paclitaxel + MANS as compared to paclitaxel alone (*n* = 5). **C.** H&E and immunohistochemical staining of phospho-MARCKS (Ser159/163), phospho-Src (Tyr416), PCNA and activated caspase-3 in xenograft tumors (*n* = 5) as described in B. Representative images are shown and positive staining is quantified (mean ± SD, *p < 0.05 versus vehicle group). T: tumor mass.

To confirm whether MARCKS inhibition by MANS peptide promotes anti-tumor activity of paclitaxel *in vivo*, MDA-MB-468 cells were injected orthotopically into the fat pads of nude mice. After tumors were palpable in all animals (see Methods), cohorts of mice were intraperitoneally injected with vehicle, MANS peptide (12.5 mg/kg), paclitaxel (3 mg/kg) alone or together with MANS peptide (12.5 mg/kg) every three days for 7 injections (*n* = 5 mice/group). Tumor size was greatly reduced in the paclitaxel plus MANS-treated group, whereas the other three groups showed continuous growth (Figure 5A). As shown in Figure 5B, the combination of MANS peptide and paclitaxel elicited a 2.6-fold growth inhibition of breast tumors, compared to the vehicle group. In contrast, treatment with either MANS peptide or paclitaxel alone resulted in marginal growth inhibition. Additionally, IHC staining showed a concomitant increase of phospho-MARCKS and phospho-Src levels in xenografted tumors receiving paclitaxel alone (Figure 5C). In the combination-treated group, levels of phospho-MARCKS and phospho-Src were reduced to 31% and 27%, respectively. Reduced proliferation and increased cell death were also observed in the combination-treated tumors by measuring abundance of PCNA, a proliferation marker; and activated caspase-3, a hallmark of apoptosis.

### Inhibition of phospho-MARCKS impairs angiogenic activity of breast cancer

**Figure 6:**
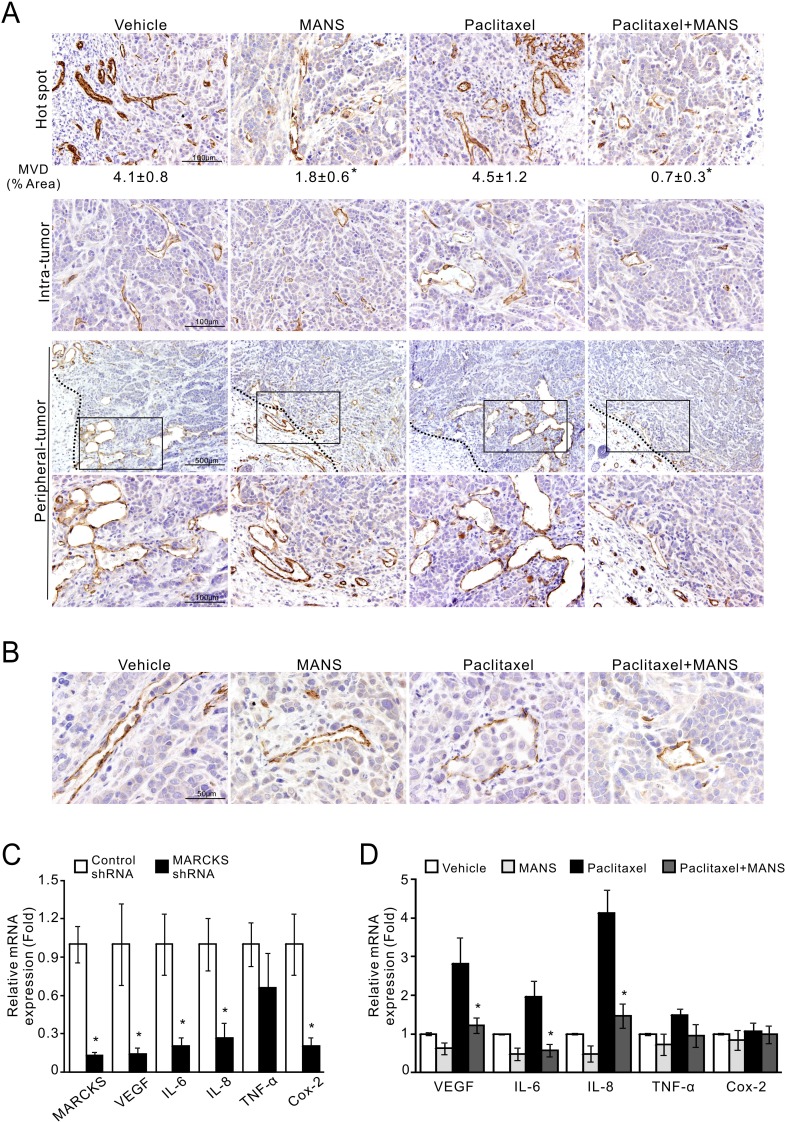
Suppression of phospho-MARCKS reduces microvessel density and the expression of angiogenic factors in TNBC cells **A-B.** MDA-MB-468 cells were injected orthotopically into female nude mice as described in Figure 5. Tissues were stained with the endothelial marker CD31. Representative pictures are shown. A, CD31-stained cells in the most vascular areas (hot spots); microvessel density (MVD) is expressed as the percentage of CD31+ area per high-powered field. Analysis was performed on 6 to 12 fields with higher magnification per tumor with ImageJ software. Data shown as mean ± SD, _*_: *p* < 0.05 as compared to paclitaxel-treated group (*top*). *Middle*, CD31-stained cells in the intra-tumor area. *Bottom*, CD31-stained cells in the periphery of tumor mass by 40X and 200X (an enlarged image from the frame) magnification. A dark dotted line represents the boundary between tumor mass and adjacent tissue. B, cancer cells within the neovasculature of vehicle- and paclitaxel-treated tumors. **C.** MDA-MB-468 cells were transduced with MARCKS-specific or non-specific shRNA-containing lentiviruses. After 72 hours of transduction, expression of angiogenic factors as indicated were determined by quantitative RT-PCR (*n* = 3, _*_: *p* < 0.05 versus control shRNA). **D.** Down-regulation of angiogenic factors in MANS-treated cells. MDA-MB-468 cells were pre-treated with 100 μM MANS peptide for 12 hours and then co-treated with or without 10nM paclitaxel. After 24 hours of co-treatment, these cells were collected and subjected to quantitative RT-PCR analyses (*n* = 3, _*_: *p* < 0.05 versus paclitaxel-treated group).

The process of new blood vessel formation is known to play a central role in supporting tumor growth and metastasis in breast cancer [32]. According to the findings in Figure 5, we postulated that tumor growth may be reduced partially through inhibition of tumor vasculature. To test this hypothesis, we assessed CD31-stained cells in these xenograft tumors and found a decrease in tumor microvessel density (MVD) in the tumors receiving paclitaxel plus MANS peptide (Figure 6A, top; mean MVD, 0.7% area). Unexpectedly, MVD in the tumors receiving MANS peptide alone was less than that in the paclitaxel-treated group (mean MVD, 1.8% area for MANS peptide alone and 4.5% area for paclitaxel alone). In addition to intra-tumor area, the combination of paclitaxel and MANS peptide treatment caused the greatest reduction in vascular number and size in the periphery of tumor mass. Of note, we found several cancer cells within the neovasculature of tumors treated with vehicle or paclitaxel alone (Figure 6B). To explore whether MARCKS inhibition interferes with the angiogenic activity of breast cancer, we examined the expression of several angiogenic factors after knockdown of MARCKS. Analysis of mRNA expression demonstrated that VEGFA, IL-6, IL-8, and Cox-2 expression in MARCKS-silenced cells were decreased to 14%, 20%, 27% and 20%, respectively (Figure 6C). As expected, a similar inhibition of angiogenic factors was seen in breast cancer cells with combination treatment of 10 nM of paclitaxel and 100 μM of MANS peptide (Figure 6D). These data suggest a potential role for MANS peptide to suppress angiogenic activity.

## DISCUSSION

A number of chemoresistance-associated signaling pathways have been identified in breast cancer. However, many of these molecules are not specific and implicated in resistance to a wide range of chemotherapeutic agents. The results of this study identified a specific target for predicting unresponsiveness of breast cancer to paclitaxel. First, elevated phospho-MARCKS abundance was positively correlated with poor differentiation and invasion/metastasis of breast tumors. Second, phospho-MARCKS abundance accumulated in response to the treatment of breast cancer cells with mitotic inhibitors, particularly paclitaxel. Lastly, knockdown of MARCKS or pharmacologic agents reduced paclitaxel-induced phospho-MARCKS and improved responsiveness of breast cancer cells to paclitaxel, both *in vitro* and *in vivo*. In addition to this anti-apoptotic effect, we also suggest a novel function of phospho-MARCKS to promote angiogenic activity, thereby tumor growth and metastasis. Collectively, these results demonstrate critical roles of phospho-MARCKS in the regulation of breast cancer malignancy and paclitaxel resistance.

Metastasis, the most common cause of death in breast cancer cases, poses substantial difficulties to treatment. The increased emphasis on individualized treatment has necessitated the identification of new biomarkers for metastasis, a goal which has not yet been fully realized [33]. Consistent with other cancers [23, 24, 27, 28], elevated phospho-MARCKS was associated with metastatic potential of breast cancer. The invasion and wound healing assays revealed an association between increased invasiveness and motility of breast cancer cells and up-regulation of MARCKS phosphorylation at the Ser159 and Ser163 sites. We then uncovered that down-regulation of phospho-MARCKS was able to reduce intravasation of cancer cells, microvessel density and angiogenic factors, hallmarks of metastatic development of breast cancer [32], in our xenograft model. These results attest the contribution of phospho-MARCKS to metastatic progression and the potential usage of phospho-MARCKS as a marker in predicting risk of metastasis in breast cancer patients.

Unequivocally, our results show that paclitaxel treatment causes a rapid increase in phospho-MARCKS abundance. We further speculate that phospho-MARCKS directly contributes to paclitaxel resistance, albeit the underlying mechanism remains to be validated. Among multiple chemotherapeutic agents used in treatment of breast cancers, only mitotic inhibitors increased phospho-MARCKS abundance. Conversely, reduction of phospho-MARCKS increased susceptibility of breast cancer cells to paclitaxel, while having a modest effect on the susceptibility to other chemotherapeutics. There are currently no biomarkers for predicting paclitaxel resistance that are applicable in a clinical setting; numerous candidates have been studied, but in most cases, no consensus has been reached [6]. Furthermore, many of the candidates studied would not be practical as specific markers for taxanes resistance; for example, P-glycoprotein, a member of the ATP-binding cassette transporter family, not only confers taxanes resistance but also participates in multiple drug resistance including resistance to anthracyclines and etoposides [34].

The major function of mitotic inhibitors is to interfere with spindle microtubule dynamics, causing cell cycle arrest and apoptosis [2, 3]. To address the possibility that persistent elevation of phospho-MARCKS predispose breast cancer cells to the treatment of paclitaxel, there are two possible mechanisms by which elevated phospho-MARCKS attenuates the anti-cancer efficacy of paclitaxel. First, phospho-MARCKS in breast cancer acts upstream Src activation, which has been documented in the regulation of paclitaxel resistance [9, 10]. This indicates that the elevation of phospho-MARCKS functions in activating Src signaling, ultimately leading to activation of cell-survival pathways to confer drug resistance. Alternatively, phospho-MARCKS could stimulate the expression of pro-inflammatory mediators, such as VEGF, IL-6 and IL-8. This is supported by results shown in Figure 6D that several messages for pro-inflammatory mediators increased in paclitaxel-treated cells. Conceivably, up-regulation of phospho-MARCKS-dependent production of cytokines, chemokines, and growth factors not only promotes inflammation events but also plays a critical role in tumor proliferation, angiogenesis, and metastasis [35-37].

During in the course of chemotherapeutic treatment, many drugs not only lose their efficacy due to chemoresistance, but also worsen the situation by contributing to metastasis themselves [38]. It has been suggested that chemotherapy-induced changes in the tumor microenvironment could attribute to its associated metastatic progression. For example, inflammation, a tumor-promoting factor, is well known to have a role in conveying the failure of therapy and promoting metastasis. Many studies have demonstrated that in response to paclitaxel administration, cancer cells up-regulate inflammatory mediators such as IL-1β, IL-6, IL-8, and VEGF-A, which also facilitate angiogenic activity [13-17]. Moreover, paclitaxel treatment can promote epithelial-mesenchymal transition, pseudopodia formation and cell migration [11]. Previously, MANS peptide, a pharmacological inhibitor of MARCKS, was shown to reduce MARCKS phosphorylation, inflammatory cytokine productions and cell motility [18, 20, 22, 27, 39]. Here, we showed that MANS peptide attenuates the effect of paclitaxel-induced phospho-MARCKS and enhances the paclitaxel sensitivity of breast cancer. In addition to blocking phospho-MARCKS-associated functions, it is possible that MANS peptide may suppress paclitaxel-induced inflammation directly or indirectly, thereby improving the anti-tumor ability of paclitaxel. The combination treatment of paclitaxel and MANS peptide demonstrated here both *in vitro* and *in vivo* studies suggests that MANS or a similar peptide that blocks paclitaxel-mediated elevation of phospho-MARCKS could be further tested in clinical trials as an adjuvant to chemotherapeutics for breast cancer.

Angiogenesis is another pathway of creating a microenvironment favorable to tumor growth and metastasis [32, 35]. In particular, paclitaxel treatment has been shown to up-regulate VEGF expression, increase mobilization of circulating endothelial cells, induce production of endothelial cell progenitors, and enhance tumor-cell homing and adhesion to endothelial cells [17, 40-42]. Although most effects by anti-angiogenic therapy on tumor expansion appeared to be transient in both preclinical and clinical settings [43], several studies have demonstrated that targeting angiogenic factors, mainly VEGF, improves the efficacy of paclitaxel in suppressing metastasis and prolonging progression-free survival [16, 44]. Consistent with these findings, we found an increase in microvessel density and angiogenic factors in paclitaxel-treated xenografted breast tumors. However, MARCKS inhibition clearly repressed angiogenic activity in both MANS-treated groups. Xenografted tumors receiving MANS alone showed a suppression of microvessel density but no tumor regression. MANS-treatment, similar to the anti-VEGF therapy [45], might prune and normalize the tumor vasculature than shrink the tumor *per se*. Previous studies have established the role of phospho-MARCKS in directing cell movement of vascular endothelial cells [46]; our results herein coupled with their findings not only confirm that up-regulation of phospho-MARCKS plays a critical role in tumor-related angiogenesis, but also suggest that phospho-MARCKS is a previously unrecognized, useful target for combination therapy regimes that seek to synergistically enhance paclitaxel efficacy by targeting angiogenesis.

TNBCs are a heterogeneous group of tumors that lack estrogen and progesterone receptors and do not over-express human epidermal growth factor receptor 2 (HER-2/neu). Unlike non-TNBCs, TNBCs tend to be highly malignant and patients with TNBCs have poor prognosis [47]. Most TNBCs are histologically classified as high grade invasive ductal carcinoma. In our clinical observations, phospho-MARCKS levels were significantly increased in invasive ductal carcinoma and high grade tumors. Actually, some of TNBC-associated molecules have been identified, such as PI3K/AKT, Src and VEGF. Unfortunately, inhibition of these signaling pathways via their inhibitors either is still under investigation or has failed in the early trials [48-50]. As our results have demonstrated, phospho-MARCKS is involved in the regulation of Src activity and VEGF expression in TNBC cells. Moreover, it has been documented that phospho-MARCKS levels function in the PI3K/AKT pathway [27, 46]. These findings indicate that phospho-MARCKS is a critical molecule in promoting malignancy in TNBCs. Indeed, our study demonstrates that elevated phospho-MARCKS augments a variety of signaling pathways and tumor behaviors, including invasion/metastasis, induction of angiogenesis and paclitaxel resistance in TNBC cell lines such as MDA-MB-231 and MDA-MB-468 cells. Accordingly, it is logical that targeting phospho-MARCKS could simultaneously attenuate several pro-inflammatory or pro-metastatic pathways in TNBCs. Due to the lack of a proven therapeutic target, treatment of TNBCs may benefit from the suppression of elevated phospho-MARCKS; and the combination therapy consisting of paclitaxel and MARCKS inhibitors may therefore aid in the development of novel treatment strategies for patients with TNBCs and other treatment refractory ER- or HER-2-positive breast cancers.

In summary, we provide evidence that mitotic inhibitors, notably paclitaxel, cause a rapid activation of phospho-MARCKS, thereby resistance to paclitaxel. The use of paclitaxel alongside with MARCKS inhibitors or molecules that can block phospho-MARCKS-mediated functions may improve paclitaxel efficacy and permit lower doses of paclitaxel to be used. Importantly, it is plausible that phospho-MARCKS serves as an informative biomarker and druggable target for paclitaxel resistance in breast cancer.

## MATERIALS AND METHODS

### Materials

All reagents, primers and antibodies used in this study are described in the Supplementary Methods.

### Cell culture and lentiviral short hairpin RNA–mediated knockdown

Breast cancer cell lines, MDA-MB-231, MDA-MB-468, and MCF7 cells were purchased from the American Type Culture Collection (ATCC) (Manassas, VA). Cells were cultured in Dulbecco's Modified Eagle's medium with 10% fetal bovine serum and 1% penicillin-streptomycin at 37°C in a humidified atmosphere of 5% CO_2_. For lentivirus-based short hairpin RNA-mediated knockdown, viruses were produced by co-transfection of HEK293T cells with the appropriate MARCKS shRNA-containing lentiviral vector and a packing DNA mix, using Lipofectamine 2000 (Invitrogen). Host cells were transduced with lentiviral constructs at three different Multiplicities of Infection (MOIs) in polybrene (8 μg/mL)-containing medium. Twenty-four hours after infection, the cells were treated with puromycin (2 μg/mL) and puromycin-resistant clones were selected and pooled.

### Patient tumor specimens and immunohistochemical staining

Two cohorts of breast tumors (which include 21 and 50 patients) were obtained from patients with histologically confirmed breast tumors who underwent surgical resection at the City of Hope National Medical Center (Duarte, CA) and UC Davis Medical Center (Sacramento, CA), respectively. This investigation was approved by the Institutional Review Board of the City of Hope National Medical Center and UC Davis Health System. Written informed consent was obtained from all patients. Formalin-fixed and paraffin-embedded specimens were used, and immunohistochemical staining was performed for phospho-MARCKS levels as well as MARCKS expression as described previously [27-29]. Detailed information on staining scoring is included in the Supplementary Methods.

### Cell invasion assay

*In vitro* cell invasion assays were performed as previously described [27] using transwell chambers (8 μm pore size; Costar, Cambridge, MA). Filters were coated with matrigel (Becton Dickinson, Franklin Lakes, NJ), and 2 × 10^4^ cells were seeded onto the matrigel. After 20 hours of incubation, filters were swabbed with a cotton swab, fixed with methanol and then stained with Giemsa solution (Sigma, St Louis, MO). The cells attached to the lower surface of the filter were counted under a light microscope.

### Scratch wound-healing assay

Cells were seeded to six-well tissue culture dishes and grown to confluence. Each confluent monolayer was then wounded linearly using a pipette tip, and washed three times with PBS. Thereafter, cell morphology and migration were observed and photographed at 16 hours. The number of cells migrating into the cell-free zone was acquired under a light microscope.

### Cell viability and colony formation assays

Trypan blue exclusion test and MTS assay were used to quantitate viable cell numbers. For Trypan blue test, cells were plated on 12-well plates and treated with the indicated chemotherapeutic agents. After 72 hours, both attached and detached cells were collected and then stained with 0.2% trypan blue (0.1% final concentration), and the number of trypan blue-positive and -negative cells was counted using a haemocytometer under low-power microscopy. For a MTS assay, cells were seeded into 96-well plates and cultured for the indicated treatment. Cell viability was evaluated by the MTS assay according to the manufacturer's protocol (Promega, Madison, WI). The absorbance measured at 490 nm was on a multi-well scanning spectrophotometer (Victor3; Perkin-Elmer, Boston, MA). For the anchorage-dependent growth assay, 200 cells were seeded in each well of 6-well plates. Cells were treated with paclitaxel and MANS peptide at the indicated concentrations for 5 days and then changed to the complete culture medium; these cells were further incubated for 5 days. Colonies were stained using 0.001% crystal violet and the number of colonies with a diameter greater than 0.5 mm was counted under an inverted microscope.

### Western blot analysis

Western blot analyses and the preparations of whole-cell lysates have been previously described [27]. Whole cell lysates were prepared by lysing cells in a lysis buffer (50 mM Tris-HCl (pH 7.4), 1% NP-40, 150 mM NaCl, 1 mM EDTA, 20 μg/ml leupeptin, 1 mM PMSF and 20 μg/ml aprotinin), and proteins were then subjected to separating by SDS-PAGE. Immunoblotting was conducted with appropriate antibodies followed by chemiluminescent detection.

### Quantitative PCR

The mRNA expression level of target genes was detected by real-time reverse transcription polymerase chain reaction (RT-PCR). The primers are described in the Supplementary Methods. We used the house keeping gene β-actin (ACTB) as the reference gene in real time RT-PCR assay. The relative expression level of target genes compared with that of ACTB was defined as –ΔCT = –[CT_target_–CT_ACTB_]. The target/ACTB mRNA ratio was calculated as 2^−ΔCT^ × K, where K is a constant.

### Xenograft models of breast cancer

Six-week-old female nude mice (supplied by The Jackson Laboratory) were housed and fed autoclaved food *ad libitum*. Detailed information on orthotopic implantation of tumors is included in the Supplementary Methods.

### Statistical analysis

Data are presented either as the mean ± SD or the mean ± SE of at least three independent experiments. The quantitative *in vitro* and *in vivo* data were analyzed using the Student's t-test. The difference in patient characteristics between the high-expression and the low-expression groups was analyzed using Fisher's exact test. All analyses were performed using SPSS software (v20.0; SPSS, Inc., Chicago, IL). All statistical tests were two-sided, and *p* values < 0.05 were considered statistically significant.

## SUPPLEMENTARY MATERIAL & METHODS, TABLE AND FIGURES



## References

[1] Cardoso F, Fallowfield L, Costa A, Castiglione M, Senkus E (2011). Locally recurrent or metastatic breast cancer: ESMO Clinical Practice Guidelines for diagnosis, treatment and follow-up. Ann Oncol.

[2] Smith JA, Wilson L, Azarenko O, Zhu X, Lewis BM, Littlefield BA, Jordan MA (2010). Eribulin binds at microtubule ends to a single site on tubulin to suppress dynamic instability. Biochemistry.

[3] McGrogan BT, Gilmartin B, Carney DN, McCann A (2008). Taxanes, microtubules and chemoresistant breast cancer. Biochim Biophys Acta.

[4] Cortes J, Vidal M (2012). Beyond taxanes: the next generation of microtubule-targeting agents. Breast Cancer Res Treat.

[5] Cobleigh MA (2011). Other options in the treatment of advanced breast cancer. Semin Oncol.

[6] Murray S, Briasoulis E, Linardou H, Bafaloukos D, Papadimitriou C (2012). Taxane resistance in breast cancer: mechanisms, predictive biomarkers and circumvention strategies. Cancer Treat Rev.

[7] Hamilton A, Hortobagyi G (2005). Chemotherapy: what progress in the last 5 years?. J Clin Oncol.

[8] Longley DB, Johnston PG (2005). Molecular mechanisms of drug resistance. J Pathol.

[9] Le XF, Bast RC (2011). Src family kinases and paclitaxel sensitivity. Cancer Biol Ther.

[10] Delle Monache S, Sanita P, Calgani A, Schenone S, Botta L, Angelucci A (2014). Src inhibition potentiates antitumoral effect of paclitaxel by blocking tumor-induced angiogenesis. Exp Cell Res.

[11] Kajiyama H, Shibata K, Terauchi M, Yamashita M, Ino K, Nawa A, Kikkawa F (2007). Chemoresistance to paclitaxel induces epithelial-mesenchymal transition and enhances metastatic potential for epithelial ovarian carcinoma cells. Int J Oncol.

[12] Meads MB, Gatenby RA, Dalton WS (2009). Environment-mediated drug resistance: a major contributor to minimal residual disease. Nat Rev Cancer.

[13] Pusztai L, Mendoza TR, Reuben JM, Martinez MM, Willey JS, Lara J, Syed A, Fritsche HA, Bruera E, Booser D, Valero V, Arun B, Ibrahim N, Rivera E, Royce M, Cleeland CS (2004). Changes in plasma levels of inflammatory cytokines in response to paclitaxel chemotherapy. Cytokine.

[14] Wang TH, Chan YH, Chen CW, Kung WH, Lee YS, Wang ST, Chang TC, Wang HS (2006). Paclitaxel (Taxol) upregulates expression of functional interleukin-6 in human ovarian cancer cells through multiple signaling pathways. Oncogene.

[15] Lee LF, Haskill JS, Mukaida N, Matsushima K, Ting JP (1997). Identification of tumor-specific paclitaxel (Taxol)-responsive regulatory elements in the interleukin-8 promoter. Mol Cell Biol.

[16] Volk LD, Flister MJ, Bivens CM, Stutzman A, Desai N, Trieu V, Ran S (2008). Nab-paclitaxel efficacy in the orthotopic model of human breast cancer is significantly enhanced by concurrent anti-vascular endothelial growth factor A therapy. Neoplasia.

[17] Kim HS, Oh JM, Jin DH, Yang KH, Moon EY (2008). Paclitaxel induces vascular endothelial growth factor expression through reactive oxygen species production. Pharmacology.

[18] Green TD, Crews AL, Park J, Fang S, Adler KB (2011). Regulation of mucin secretion and inflammation in asthma: a role for MARCKS protein?. Biochim Biophys Acta.

[19] Singer M, Martin LD, Vargaftig BB, Park J, Gruber AD, Li Y, Adler KB (2004). A MARCKS-related peptide blocks mucus hypersecretion in a mouse model of asthma. Nat Med.

[20] Eckert RE, Neuder LE, Park J, Adler KB, Jones SL (2010). Myristoylated alanine-rich C-kinase substrate (MARCKS) protein regulation of human neutrophil migration. Am J Respir Cell Mol Biol.

[21] Park JA, Crews AL, Lampe WR, Fang S, Park J, Adler KB (2007). Protein kinase C delta regulates airway mucin secretion via phosphorylation of MARCKS protein. Am J Pathol.

[22] Li J, D'Annibale-Tolhurst MA, Adler KB, Fang S, Yin Q, Birkenheuer AJ, Levy MG, Jones SL, Sung EJ, Hawkins EC, Yoder JA, Nordone SK (2013). A myristoylated alanine-rich C kinase substrate-related peptide suppresses cytokine mRNA and protein expression in LPS-activated canine neutrophils. Am J Respir Cell Mol Biol.

[23] Rombouts K, Carloni V, Mello T, Omenetti S, Galastri S, Madiai S, Galli A, Pinzani M (2013). Myristoylated Alanine-Rich protein Kinase C Substrate (MARCKS) expression modulates the metastatic phenotype in human and murine colon carcinoma *in vitro* and *in vivo*. Cancer Lett.

[24] Techasen A, Loilome W, Namwat N, Takahashi E, Sugihara E, Puapairoj A, Miwa M, Saya H, Yongvanit P (2010). Myristoylated alanine-rich C kinase substrate phosphorylation promotes cholangiocarcinoma cell migration and metastasis via the protein kinase C-dependent pathway. Cancer Sci.

[25] Yang Y, Chen Y, Saha MN, Chen J, Evans K, Qiu L, Reece D, Chen GA, Chang H (2015). Targeting phospho-MARCKS overcomes drug-resistance and induces antitumor activity in preclinical models of multiple myeloma. Leukemia.

[26] Browne BC, Hochgrafe F, Wu J, Millar EK, Barraclough J, Stone A, McCloy RA, Lee CS, Roberts C, Ali NA, Boulghourjian A, Schmich F, Linding R, Farrow L, Gee JM, Nicholson RI (2013). Global characterization of signalling networks associated with tamoxifen resistance in breast cancer. FEBS J.

[27] Chen CH, Thai P, Yoneda K, Adler KB, Yang PC, Wu R (2014). A peptide that inhibits function of Myristoylated Alanine-Rich C Kinase Substrate (MARCKS) reduces lung cancer metastasis. Oncogene.

[28] Chen CH, Chiu CL, Adler KB, Wu R (2014). A novel predictor of cancer malignancy: up-regulation of myristoylated alanine-rich C kinase substrate phosphorylation in lung cancer. Am J Respir Crit Care Med.

[29] Chen CH, Statt S, Chiu CL, Thai P, Arif M, Adler KB, Wu R (2014). Targeting myristoylated alanine-rich C kinase substrate phosphorylation site domain in lung cancer. Mechanisms and therapeutic implications. Am J Respir Crit Care Med.

[30] Reddy SP, Natarajan V, Dudek AZ (2014). MARCKS Is Marked in Combating Lung Cancer Growth and Acquired Resistance. Am J Respir Crit Care Med.

[31] Gordon LA, Mulligan KT, Maxwell-Jones H, Adams M, Walker RA, Jones JL (2003). Breast cell invasive potential relates to the myoepithelial phenotype. Int J Cancer.

[32] Schneider BP, Miller KD (2005). Angiogenesis of breast cancer. J Clin Oncol.

[33] Weigelt B, Peterse JL, van ‘t Veer LJ (2005). Breast cancer metastasis: markers and models. Nat Rev Cancer.

[34] Martin HL, Smith L, Tomlinson DC (2014). Multidrug-resistant breast cancer: current perspectives. Breast Cancer (Dove Med Press).

[35] Hanahan D, Weinberg RA (2011). Hallmarks of cancer: the next generation. Cell.

[36] Finger EC, Giaccia AJ (2010). Hypoxia, inflammation, and the tumor microenvironment in metastatic disease. Cancer Metastasis Rev.

[37] Colotta F, Allavena P, Sica A, Garlanda C, Mantovani A (2009). Cancer-related inflammation, the seventh hallmark of cancer: links to genetic instability. Carcinogenesis.

[38] Vyas D, Laput G, Vyas AK (2014). Chemotherapy-enhanced inflammation may lead to the failure of therapy and metastasis. Onco Targets Ther.

[39] Green TD, Park J, Yin Q, Fang S, Crews AL, Jones SL, Adler KB (2012). Directed migration of mouse macrophages *in vitro* involves myristoylated alanine-rich C-kinase substrate (MARCKS) protein. J Leukoc Biol.

[40] Shaked Y, Henke E, Roodhart JM, Mancuso P, Langenberg MH, Colleoni M, Daenen LG, Man S, Xu P, Emmenegger U, Tang T, Zhu Z, Witte L, Strieter RM, Bertolini F, Voest EE (2008). Rapid chemotherapy-induced acute endothelial progenitor cell mobilization: implications for antiangiogenic drugs as chemosensitizing agents. Cancer Cell.

[41] Roodhart JM, Langenberg MH, Vermaat JS, Lolkema MP, Baars A, Giles RH, Witteveen EO, Voest EE (2010). Late release of circulating endothelial cells and endothelial progenitor cells after chemotherapy predicts response and survival in cancer patients. Neoplasia.

[42] Daenen LG, Roodhart JM, van Amersfoort M, Dehnad M, Roessingh W, Ulfman LH, Derksen PW, Voest EE (2011). Chemotherapy enhances metastasis formation via VEGFR-1-expressing endothelial cells. Cancer Res.

[43] Bergers G, Hanahan D (2008). Modes of resistance to anti-angiogenic therapy. Nat Rev Cancer.

[44] Miller K, Wang M, Gralow J, Dickler M, Cobleigh M, Perez EA, Shenkier T, Cella D, Davidson NE (2007). Paclitaxel plus bevacizumab versus paclitaxel alone for metastatic breast cancer. N Engl J Med.

[45] Jain RK, Duda DG, Willett CG, Sahani DV, Zhu AX, Loeffler JS, Batchelor TT, Sorensen AG (2009). Biomarkers of response and resistance to antiangiogenic therapy. Nat Rev Clin Oncol.

[46] Kalwa H, Michel T (2011). The MARCKS protein plays a critical role in phosphatidylinositol 4,5-bisphosphate metabolism and directed cell movement in vascular endothelial cells. J Biol Chem.

[47] Engebraaten O, Vollan HK, Borresen-Dale AL (2013). Triple-negative breast cancer and the need for new therapeutic targets. Am J Pathol.

[48] Criscitiello C, Azim HA, Schouten PC, Linn SC, Sotiriou C (2012). Understanding the biology of triple-negative breast cancer. Ann Oncol.

[49] Guarneri V, Dieci MV, Conte P (2013). Relapsed triple-negative breast cancer: challenges and treatment strategies. Drugs.

[50] Finn RS, Bengala C, Ibrahim N, Roche H, Sparano J, Strauss LC, Fairchild J, Sy O, Goldstein LJ (2011). Dasatinib as a single agent in triple-negative breast cancer: results of an open-label phase 2 study. Clin Cancer Res.

