# Supplementary material for: Elevated MARCKS phosphorylation contributes to unresponsiveness of breast cancer to paclitaxel treatment
Source: Oncotarget. 2015 Apr 14;6(17):15194–208. doi: 10.18632/oncotarget.3827 (PMC4558145; doi:10.18632/oncotarget.3827)
Supplement: Supplementary file 1 [file oncotarget-06-15194-s001.pdf]

Elevated MARCKS phosphorylation contributes to unresponsiveness of breast cancer to paclitaxel treatment

Supplementary Material & Methods

Reagents and antibodies

Dulbecco's Modified Eagle's medium, fetal bovine serum and penicillin-streptomycin were purchased from Life Technologies Inc. (Carlsbad, CA). Lipofect-AMINE™ was purchased from Invitrogen (Carlsbad, CA). VECTASTAIN® Elite ABC Kit (Rabbit IgG), VECTOR® Hematoxylin QS nuclear counterstain and DAB solution were purchased from VECTOR Laboratories Inc. (Burlingame, CA). Anti-CD 31 was purchased from Abcam (Cambridge, MA). Both anti-pSer159/163 MARCKS (clone EP2113Y) and anti-MARCKS (clone EP1446Y) were purchased from Epitomics (Burlingame, CA). Anti-pSer159/163 MARCKS (clone D13D2), anti-pSer473 AKT, anti-AKT, anti-pY416 Src, anti-Src, anti-cleaved PARP, anti-cleaved caspase 3, anti-PARP, anti-caspase 3, anti-PCNA and anti-β-actin antibodies were purchased from Cell Signaling Technology, Inc. (Danvers, MA). MARCKS siRNAs (MARCKS siRNA Smartpool) and DharmaFECT siRNA transfection reagents were purchased from Dharmacon, Inc. (Lafayette, CO). Rottlerin, Gö 6976, εV1-2 and PKCß inhibitor were purchased from Calbiochem-EMD Millipore (Chicago, IL).

Plasmid constructs and primers

For generation of MARCKS shRNA plasmids, the oligonucleotide of shRNAs (shRNA: 5'-GAGCGCTTCTCCTTCAAGAA-3' and its complementary strand: 5'-TTCTTGAAGGAGAAGCGCTC-3') were synthesized, annealed and cloned into the pGreenPuro shRNA expression lentivector (System Biosciences, Mountain View, CA). The V5-tagged wild type and mutant MARCKS were expressed in mammalian cells by

using pcDNA3.1-wild type MARCKS and pcDNA3.1-S159/163A MARCKS constructs which have been previously described in detail [1]. The all primers for quantitative real-time PCR used were as follows: the VEGFA forward primer 5'-CCTTGCTGCTCTACCTCCA-3' and the reverse primer 5'-CAAATGCTTTCTCCGCTCT-3'; the TNF-α forward primer 5'-CTGGAAAGGACACCATGAGCACT-3' and the reverse primer 5'-TTGATGGCAGAGAGGAGGTTGAC-3'; the IL-6 forward primer 5'-TGACAAACAAATTCGGTACATCCT-3' and the reverse primer 5'-AGTGCCTCTTTGCTGCTTTCAC-3'; the IL-8 forward primer 5'-ACATGACTTCCAAGCTGGCCGTGG-3' and the reverse primer 5'-GTATGTTCTGGATATTTCATGGTAC-3'; the Cox-2 forward primer 5’-ATCATTCACCAGGCAAATTGC-3’ and the reverse primer 5’-GGCTTCAGCATAAAGCGTTTG-3'; the MARCKS forward primer 5′-TTGTTGAAGAAGCCAGCATGGGTG-3′ and the reverse primer 5′-TTACCTTCACGTGGCCATTCTCCT-3.

Patient tumor specimens and immunohistochemical staining

Two cohorts of breast tumors (which include 21 and 50 patients) were obtained from patients with histologically confirmed breast tumors who underwent surgical resection at the City of Hope National Medical Center (Duarte, CA) and UC Davis Medical Center (Sacramento, CA), respectively. This investigation was approved by the Institutional Review Board of the City of Hope National Medical Center and UC Davis Health System. Written informed consent was obtained from all patients. Formalin-fixed and paraffin-embedded specimens were used, and immunohistochemical staining was performed for phospho-MARCKS levels as well as MARCKS expression. Detailed experimental procedures were modified from the

paraffin immunohistochemistry protocol supplied by the manufacturer (Cell Signaling, Danvers, MA). The slides were de-paraffinized in xylene and rehydrated in graded alcohol and water. An antigen retrieval step (10 nM sodium citrate (pH 6.0) at a sub-boiling temperature) was used for each primary antibody. Endogenous peroxidase activity was blocked by 3% hydrogen peroxide followed by blocking serum and incubation with appropriate antibodies overnight at 4°C. Detection of immunostaining was carried out by using the VECTASTAIN® ABC system, according to the manufacturer's instructions (Vector Laboratories, Burlingame, CA). A four-point staining intensity scoring system was devised to confirm the relative expression of phospho-MARCKS in cancer specimens; scores ranged from zero (no expression) to 3 (highest-intensity staining) as described previously [1-4]. The results were classified into two groups according to the intensity and extent of staining: in the low-expression group, staining was observed in 0–1% of the cells (staining intensity score = 0), in less than 10% of the cells (staining intensity score =1), or in 10%-25% of the cells (staining intensity score = 2); in the high-expression group, staining was present more than 25% of the cells (staining intensity score = 3).

Xenograft models of breast cancer

Six-week-old female nude mice (supplied by The Jackson Laboratory, Sacramento, CA) were housed four mice per cage and fed autoclaved food *ad libitum*. The fat pads of nude mice were injected orthotopically with 5×10^6 MDA-MB-468 cells and these mice were examined every 3 days for tumor size. Groups were randomized and treatment started when tumor size reached 100 mm^3 (the tumor volume was calculated by using the formula $V = 0.5Xab^2$, where a and b are the longest and shortest diameters of the tumors, respectively). These nude mice bearing subcutaneous tumors were intraperitoneally (*i. p.*) injected with vehicle, MANS

peptide (12.5 mg/kg), paclitaxel (3 mg/kg) alone or MANS peptide (12.5 mg/kg) combined with paclitaxel (3 mg/kg) every three days for 7 injections (n=5 mice/group). After 21 days of treatment, these mice were sacrificed and the xenografted tumors were collected for histological analysis. Mouse experiments were approved by the Institutional Animal Care and Use Committee of UC Davis.

Supplementary Data

Table S1 Summary of clinicopathologic features according to phospho-MARCKS level and MARCKS expression.

**Characteristic**	**Low**<br>No. of Patients (%)	**High**<br>No. of Patients (%)	***p* value**
**phospho-MARCKS**			
**Number of patients**	n=14	n=36	
**Tumor status**			0.048†
Benign	3 (6)	1 (2)	
Ductal carcinoma in situ	2 (4)	1 (2)	
Invasive ductal/lobular carcinoma	8 (16)	27 (54)	
Lymph node metastases	1 (2)	7 (14)	
**Grade***			0.005†
G1	3 (7)	1 (2)	
G2	4 (10)	8 (20)	
G3	2 (5)	23 (56)	
**MARCKS**			
**Number of patients**	n=8	n=42	
**Tumor status**			0.578†
Benign	1 (2)	3 (6)	
Ductal carcinoma in situ	1 (2)	2 (4)	
Invasive ductal/lobular carcinoma	5 (10)	30 (60)	
Lymph node metastases	1 (2)	7 (14)	
**Grade***			0.402†
G1	0 (0)	4 (10)	
G2	3 (7)	9 (22)	
G3	3 (7)	22 (54)	

† Fisher's exact test.

*Some patients without grade information

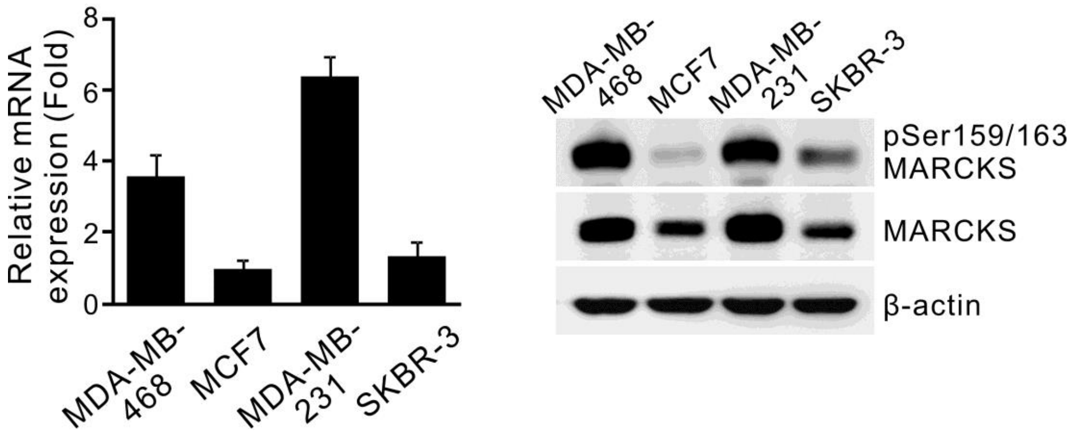

Figure S1. High expression of MARCKS and its phosphorylated form in triple negative breast cancer (TNBC) cell lines. *Left*, cells from near-confluent cultures were harvested for RNA isolation and the level of expression was quantified with quantitative RT-PCR and normalized with the β-actin level. *Right*, MARCKS protein and its Ser159/163 phosphorylation were confirmed by Western blot.

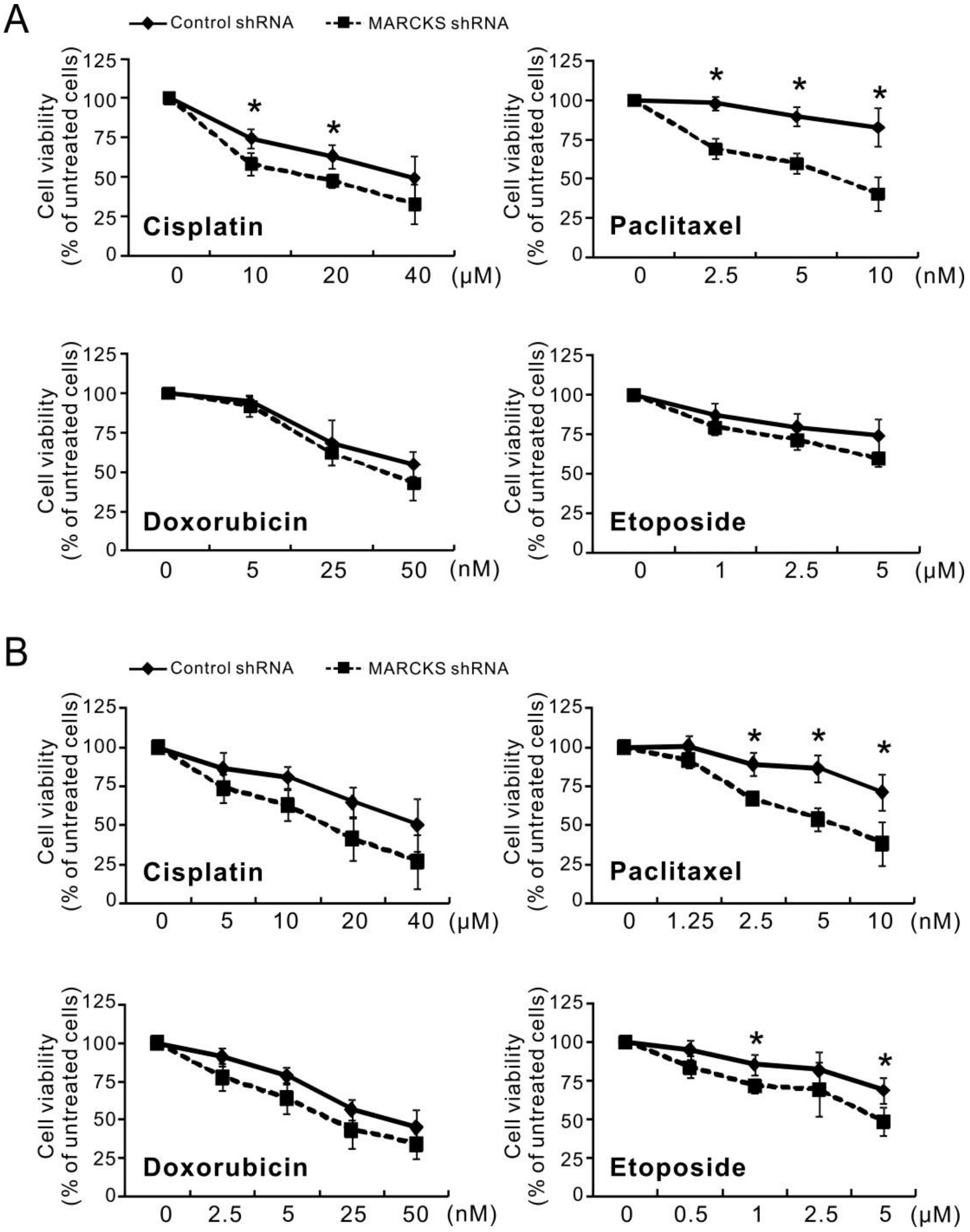

Figure S2. Effect of MARCKS inhibition on chemotherapeutic cytotoxicity. (A-B) Knockdown of MARCKS to down-regulate phospho-MARCKS levels decreases cell viability of MDA-MB-231 **(A)** and MDA-MB-468 **(B)** cells in response to chemotherapeutic agents. Cells were infected with control non-specific or MARCKS-specific shRNA-containing lentivirus. These cells were subjected to various doses of cisplatin, paclitaxel, doxorubicin or etoposide for treatment, as indicated. After 72 hours of treatment, cell viability was determinate by MTS assays. Data shown as mean± SD; *: $p < 0.05$ versus control shRNA (n=4).

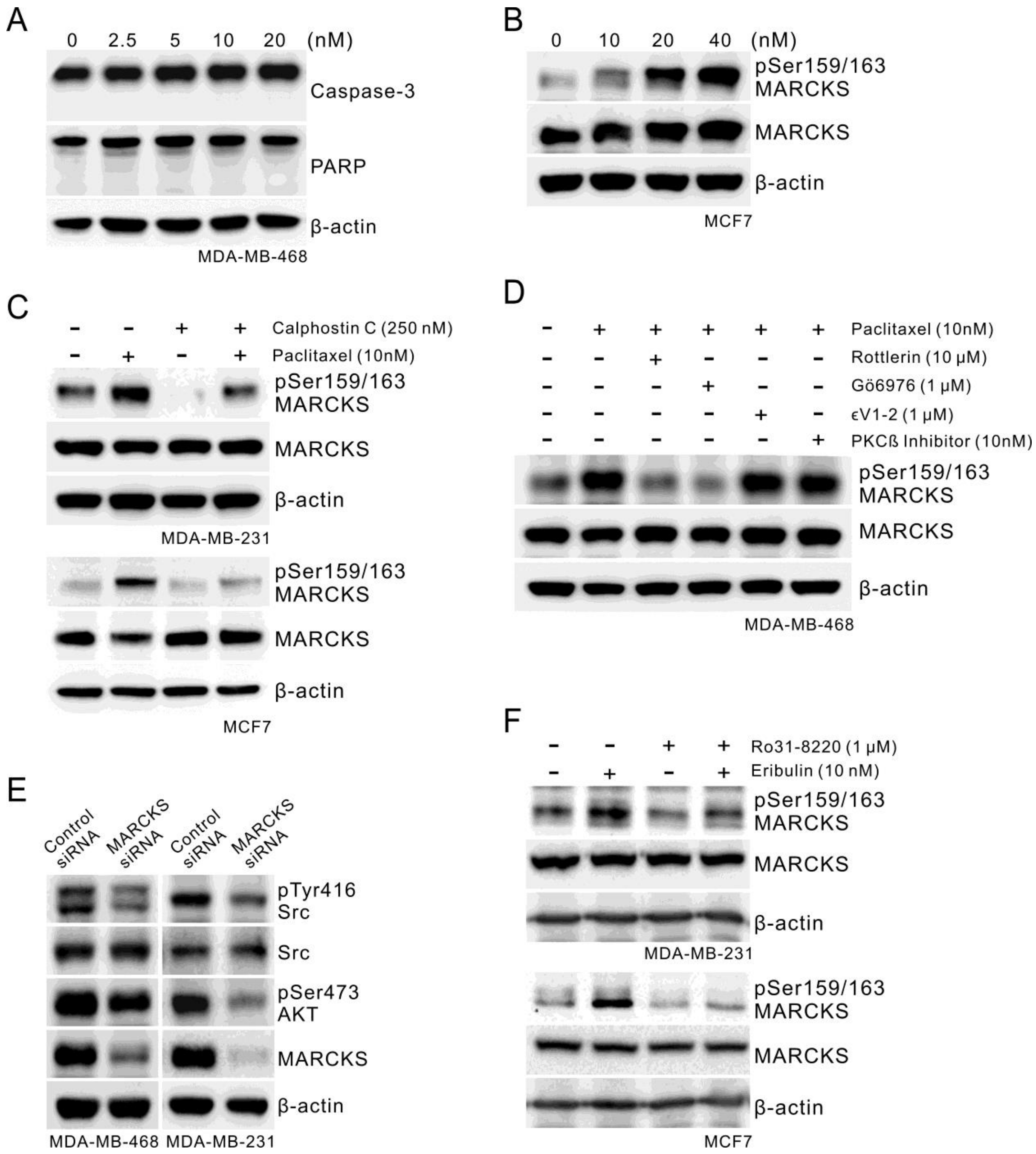

Figure S3. Increased phospho-MARCKS in mitotic inhibitors-treated cells. (A) Confirmation of apoptotic effect in paclitaxel-treated cells by using Western blot analysis. **(B)** MARCKS phosphorylaiton was induced in MCF-7 cells after paclitaxel treatment. **(C)** Inhibition of PKC activity repressed MARCKS activation in breast cancer cells in response to paclitaxel. MDA-MB-231 (*top*) and MCF-7 (*bottom*) cells were co-treated with paclitaxel (10 nM) and PKC inhibitor (Calphostin C; 250nM) for 24 hours and the cells were subjected to Western blots to assess MARCKS and its Ser159/163 phosphorylated molecule. **(D)** The effects of specific PKC isoforms

inhibitors on phospho-MARCKS abundance in paclitaxel-treated cells. Cells were co-treated with paclitaxel (10 nM) and various PKC isoforms inhibitors as indicated. The levels of phospho-MARCKS and MARCKS in the cells were determined by Western blots. Rottlerin: PKC-delta inhibitor; Gö6976: PKC-alpha inhibitor; ϵV1-2: PKC-epsilon inhibitor; PKCß: PKC-beta inhibitor. **(E)** siRNA knockdown of MARCKS expression reduced Src activity. TNBC cells were transfected with control or MARCKS-specific siRNAs. After 72 hours of transfections, lysates from these cells were subjected to Western blots. **(F)** Western blot analysis of phospho-MARCKS levels in MDA-MB-231 (*top*) and MCF-7 (*bottom*) cells with combination treatment of eribulin (10 nM) and PKC inhibitor (Ro 31-8220; 1μM) for 24 hours.

Supplementary References

1. Chen CH, Statt S, Chiu CL, Thai P, Arif M, Adler KB and Wu R. Targeting myristoylated alanine-rich C kinase substrate phosphorylation site domain in lung cancer. Mechanisms and therapeutic implications. Am J Respir Crit Care Med. 2014; 190(10):1127-1138.
2. Lu J, Guo H, Treekitkarnmongkol W, Li P, Zhang J, Shi B, Ling C, Zhou X, Chen T, Chiao PJ, Feng X, Seewaldt VL, Muller WJ, Sahin A, Hung MC and Yu D. 14-3-3zeta Cooperates with ErbB2 to promote ductal carcinoma in situ progression to invasive breast cancer by inducing epithelial-mesenchymal transition. Cancer Cell. 2009; 16(3):195-207.
3. Kuo TC, Tan CT, Chang YW, Hong CC, Lee WJ, Chen MW, Jeng YM, Chiou J, Yu P, Chen PS, Wang MY, Hsiao M, Su JL and Kuo ML. Angiopoietin-like protein 1 suppresses SLUG to inhibit cancer cell motility. J Clin Invest. 2013; 123(3):1082-1095.
4. Chen CH, Chiu CL, Adler KB and Wu R. A novel predictor of cancer malignancy: up-regulation of myristoylated alanine-rich C kinase substrate phosphorylation in lung cancer. Am J Respir Crit Care Med. 2014; 189(8):1002-1004.
